# Supplementary material for: Polyketide synthase gene clusters in the ericoid mycorrhizal fungus Oidiodendron maius: genome-wide prediction, expression profiling, and functional characterization of knockout mutants
Source: Front Plant Sci. 2026 Sep 16;17:1924430. doi: 10.3389/fpls.2026.1924430 (PMC13623903; doi:10.3389/fpls.2026.1924430)
Supplement: Supplementary file 1 [file SupplementaryFile1.zip › Supplementary Materials/Supplementary Folder 1.zip!/Oidma1_AssemblyScaffolds_Repeatmasked/knownclusterblast/cluster40/jgi.p_Oidma1_106254_mibig_hits.html]

| MIBiG Protein | Description | MIBiG Cluster | MiBiG Product | % ID | % Coverage | BLAST Score | E-value |
| --- | --- | --- | --- | --- | --- | --- | --- |
| CAH55660.1 | putative\_copper\_transporting\_P-type\_ATPase\_efflux\_pump | BGC0000259 | Prodigiosin\_biosynthetic\_gene\_cluster | 30 | 76.4331210191 | 280 | 8e-75 |
| AAY28209.1 | unknown | BGC0000074 | Herbimycin\_biosynthetic\_gene\_cluster | 28 | 77.8889899909 | 249 | 2e-65 |
| ALD82534.1 | heavy\_metal\_translocating\_P-type\_ATPase | BGC0001212 | Nannocystin\_biosynthetic\_gene\_cluster | 27 | 55.9599636033 | 133 | 1.2e-30 |
| AAY93339.1 | magnesium-importing\_ATPase | BGC0000413 | Pyoverdine\_biosynthetic\_gene\_cluster | 23 | 56.414922657 | 71 | 9.7e-12 |
